# Information Delayering Safety Management (IDSM): A New Method of System Safety in Urgent Situations Needs to Be Established

**DOI:** 10.3390/ijerph20043122

**Published:** 2023-02-10

**Authors:** Yu Lei, Guirong Zhang, Xiuping Liao, Wei Feng

**Affiliations:** 1 School of Public Administration, Central South University, Changsha 410083, China; 2 Center for Social Stability Risk Assessment, Central South University, Changsha 410083, China; 3 School of Engineering, Macquarie University, Sydney 2109, Australia

**Keywords:** system safety, safety management method, information distortion, delayering mode

## Abstract

Organizational safety decisions rely heavily on safety information in today’s data-driven era, but there is a significant danger of information distortion that can compromise system safety. To address the issue of information distortion and enhance system safety, a new approach called information delayering safety management (IDSM) has been developed and implemented. The IDSM method combines delayering management mode and graph theory to study the relationship between information distortion management and delayering management. By using the delayering mode as a theoretical foundation for safety information management, information distortion can be reduced. The implementation of this approach from a graph theory perspective has been tested using a case study and has been proven to effectively enhance the reliability of safety information and ensure system safety. The minimum control set of the directed graph algorithm can be used to realize the whole network management of safety information distortion. The amount of safety information and signal noise can be controlled by adjusting connectivity, and safety information distortion can be regulated through the adjustment of structural holes and flow direction. Overall, IDSM offers a new, effective method for accident analysis and safety management, allowing safety professionals to make informed decisions based on robust advanced evidence.

## 1. Introduction

### 1.1. Research Scope and Significance

Safety information is an important foundation for safety management and emergency response, as it plays a key role in ensuring system safety. However, the flow of safety information is often distorted, which contributes to a large proportion of rumor incidents due to public safety needs [1]. This distortion of safety information is a critical factor that can result in incorrect emergency responses and pose a threat to public safety. The breakdown of the flow of information is a potential cause of accidents [2]. Distortion of safety information flow poses a threat to system safety. Negative signal noise is important accident causation based on safety information cognition [3]. Distorted safety information flow can cause emergency response and emergency linkage to fail. In addition, safety information flow distortion can result in the public’s misunderstanding of the truth of safety incidents, producing unnecessary risks and consequences. To maintain system safety and ensure accurate emergency responses, it is essential to minimize safety information distortion and ensure the flow of accurate safety information.

Safety information distortion management is an important way to ensure system safety [4]. On the one hand, safety information distortion management is an important accident control method [2]. Safety information management is conducive to system safety and risk control. The development process and information of safety information flow distortion can be purposefully controlled through safety information management based on the delayering mode [5,6]. It helps curb the negative impact of safety information flow distortion. Information delayering safety management (IDSM) can reduce information deviation via safety information flow and by reducing safety rumors. It can ensure an accurate understanding of system safety status and, to a certain extent, the scientificity and effectiveness of crisis decision making. On the other hand, it can enhance personal interpretation and understanding of safety information, reduce the uncertainty of personal safety perception, and facilitate accident management and investigation. Adequate IDSM is key in emergency management. Real safety information should be more easily obtained and understood by the public; it can help maintain public safety and social stability. The public can then better understand the truth of safety incidents and can effectively master emergency response methods, which can be part of a “safety education curriculum” [7]. IDSM can help to obtain the understanding and support of society as a whole by releasing accurate safety information, thus offering a strong guarantee for safety management. Avoiding safety information distortion has become a key issue in safety management in the era of big data.

### 1.2. Related Work Review

Crisis management emphasizes the importance of information dissemination and risk communication [8]. Risk information dissemination plays a crucial role in crisis response [9,10]. Moreover, it has a great effect on scientific decision making [11], policy implementation [12], weather warnings [13], personal health [14], etc. An effective strategy requires a method of dissemination based on effective and reliable information [15]. However, many factors can make risk information communication difficult [16,17], such as the transmitter and receiver. If the process of risk information communication is distorted, it will result in serious consequences [18,19]. In a case where risk information communication fails, food will likely be stigmatized with a series of corporate bankruptcies and industry shocks [20].

Therefore, the distortion of risk information is an urgent problem to be solved. The literature search showed that much research has been conducted on risk communication, mainly focusing on its definition, paradigms, and management methods [21,22,23]. Existing risk information management methods are diverse, including the format of risk communication messages, the use of risk comparisons, audience differences, and the use of mental models [24]. Ancker et al. designed features of graphs to improve communication regarding quantitative health risks [25]. Keller et al. believed that effect is important in successful risk communication [26]; it can improve the recipient’s understanding regarding the following of certain basic principles when introducing risk information [27]. Frewer believed that attention should be paid to the risk of public concern [28]. Doctors must build trust with patients during risk communication. When communicating medical information and advice to patients, healthcare providers and health educators should use clear and simple language, pictograms, and clearly stated risks [29,30].

Meanwhile, research on safety information distortion has been ongoing. On the one hand, the distortion of safety information affects the occurrence of accidents. The role of safety information flow in accident causation is profound [31]. In safety management, it is important to make effective safety decisions based on reliable and sufficient safety-related information; however, many safety management failures occur due to the lack of required safety-related information for safety decisions [32]. Furthermore, failures at each level of the subsystem or between two adjacent levels are potential causes of accidents [2]. The loss of safety information transmission is a key factor leading to accidents. When the loss of safety information affects decision making, the possibility of accidents increases [33]. A prediction–decision–execution accident model is proposed, which considers that the cause of an accident can be attributed to the failure of safety information acquisition, analysis, and utilization [31]. On the other hand, the root causes of the lack of safety information can be roughly attributed to the lack of information literacy [34], human physical and mental load [35], and information sources and channels [4]. To reduce the impact of safety information shortage on safety management, two approaches have been proposed: evidence-based safety management and precise safety management. Evidence-based safety management aims to find the best evidence related to specific safety management issues [32]. Precise safety management focuses on using sufficient and accurate safety information [36].

In summary, most studies on risk information communication emphasize the influence of information sources, receivers, and channels on information distortion, while research on safety information distortion is carried out on its effect and influencing factors. However, there are still research gaps: (1) Most existing research focuses on the negative effects of safety information distortion on safety management but there is limited research on how to effectively reduce this distortion. As a result, controlling accidents caused by information distortion remains a challenge. (2) Information distortion management is typically discussed from a single perspective of safety information or signal noise, leading to low management efficiency. (3) Most research on controlling information distortion focuses on the source, receiver, and channel, without considering the impact of information structure on information distortion caused by the interaction of these factors. By adjusting the information structure, information distortion can be managed with lower costs. (4) Current methods for managing information distortion are mostly theoretical, lacking practical feasibility and the ability to be quantitatively measured.

### 1.3. Research Goals and Methods

The research problem of this paper is how to reduce information distortion to ensure system safety. The academic community generally agrees with the high information flow characteristics of the delayering model because it can improve the speed and accuracy of information. However, its application scope is mostly aimed at enterprise performance management and no method has been proposed to use the delayering model to improve the efficiency of safety management [4,37]. Existing research has not looked into the feasibility of implementing this in safety information management. How to achieve it is also an ongoing topic of existing research.

To address this problem, this paper proposes a solution based on the delayering management mode and the perspective of information structure. The goal is to reduce information distortion by controlling signal noise and improving the flow of safety information. The proposed solution is made possible through the use of graph theory. This study focuses on safety information flow distortion, with the delayering management mode as the theoretical basis and graph theory as the methodology.

**Figure 1:**
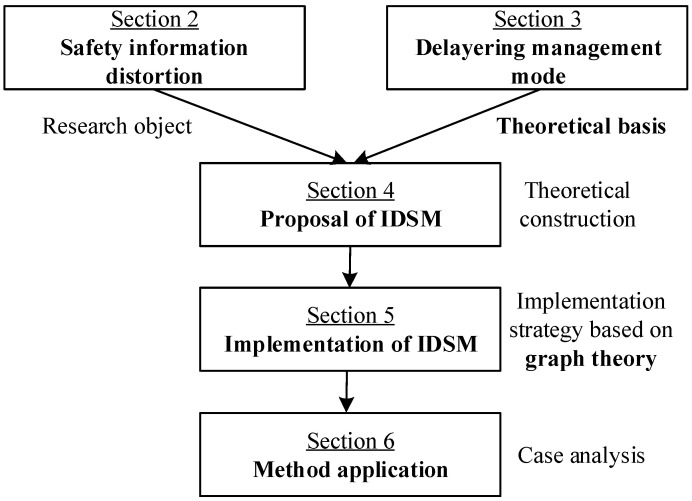
Workflow diagram of this study.

The research process is divided into seven sections in this paper. In Figure 1, the basic concepts of safety information distortion are analyzed in Section 2 and the delayering management mode is introduced in Section 3. Section 4 proposes IDSM based on Section 2 and Section 3. IDSM is implemented in Section 5. IDSM is applied to a case study in Section 6. Finally, Section 7 provides the discussion and conclusions.

## 2. Safety Information Distortion

### 2.1. Basic Concepts

Safety information distortion is a phenomenon where safety information between people deviates greatly from real safety incidents [4]. The spread of safety information distortion is a process wherein real safety incidents are distorted and false safety information is disseminated. There are mainly two opposite types of information: safety information and signal noise. The characteristics of safety information distortions are analyzed to gain a deeper understanding of safety information distortion:

(1) Deviation: Safety information distortions mask the real situation of safety incidents using misinterpretation, grafting, fabrication, one-sided magnification, etc. They are the product of safety incident distortion and therefore exhibit a deviation characteristic similar to distortion.
(2) Hazard: Safety information distortions can have a certain negative impact on public mentality and social stability, raising people’s tension, panic, uneasiness, etc. Furthermore, safety information distortions may cause public misconceptions about the victims of an incident, causing extra harm and impacting their lives. Therefore, it is crucial to study how safety information distortions can be properly quashed/dispelled and how negative effects can be avoided.
(3) Controllability [38]: Safety information distortions can be managed and suppressed in certain ways, such as suppressing its release time, contact rate, interaction rate, and transmission rate, and controlling the release time, speed, content, transmission mode, and intensity of safety information.

Safety information distortion is widely disseminated because of the public concern about safety incidents and the lack of safety information. The spread of safety information distortion can easily affect the people involved, causing psychological and physical harm, such as deception, panic, and language violence. Typical examples of safety information distortion are as follows: the “salt grabbing” phenomenon after the radiation leakage in Japan [39], the 2015 Tianjin blast rumor [40], and the explosion rumor accident in Xiangshui County [4].

Safety information distortion is largely caused by the interference of signal noise, which is false information or misinformation generated based on safety incidents. It results from a lack of safety information [3], implying that signal noise is the opposite of safety information, which is the correct information related to a safety incident, while signal noise, in this study, is false information about a safety incident. The identification or detection of rumor nodes is a crucial issue. Rumors can be traced and identified through accident analysis and investigation. The likelihood of a node becoming a rumor node can be evaluated based on the mastery of safety knowledge before an accident occurs. Key monitoring objects can be determined by sorting to assess the possibility of becoming a rumor node in safety management practices. At the same time, the information flow structure of the monitoring object is adjusted to reduce the spread of rumors, enhance the flow of safety information, and achieve accident prevention.

Certain concepts must be defined to clarify the management of safety information distortion according to graph theory. See Table 1 for details.

### 2.2. Calculation of Signal Noise Amount

**Figure 2:**
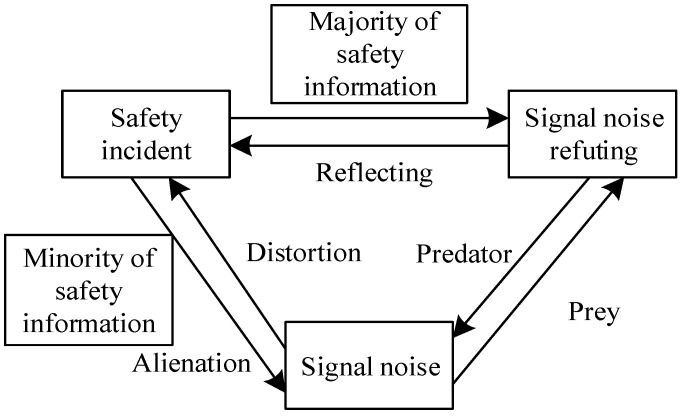
Relationship between safety information distortion and other related concepts.

Safety information and signal noise are opposing concepts; the former reflects the truth of safety incidents, whereas the latter distorts facts, as shown in Figure 2. Signal noise is transmitted along with safety information, and the dissemination of authoritative safety information can suppress the spread of signal noise. Because of the high reliability of authoritative safety information, the public is more likely to believe authoritative safety information, while the false signal noise that masks safety information is filtered out.

Therefore, the relationship between signal noise refuting and signal noise is like that between a predator and its prey: on the one hand, rumor nodes compete with refuting nodes, meaning that both types of nodes simultaneously strive for support from other nodes; on the other hand, refuting nodes are highly credible and authoritative; thus, other nodes are more likely to support refuting nodes. Therefore, refuting nodes can seize the position of rumor nodes, presenting a relationship between predator and prey [42]. IDSM can be achieved by suppressing signal noises and boosting the strength of authoritative safety information. The truth is the most powerful approach for rebuffing signal noise from a certain perspective. Therefore, an effective way of limiting the scope and impact of signal noise is by setting up refuting nodes.

The ambiguous degree of safety information directly affects the generation of signal noise to a certain extent. In 1947, Allport and Postman proposed the formula of rumor [43]:

*R_i_* = *M_i_* × *A_i_* (1)

where *R_i_* represents rumor, *M_i_* is the importance degree of the incidents, and *A_i_* is the ambiguity of the incidents.

Equation (1) reveals that the risk of the rumor is proportional to the importance degree and ambiguity of safety incidents. Because the importance degree of safety incidents is not controllable, IDSM in this study aims to bring the ambiguous degree of safety information closest to zero, so that the possibility of safety information distortion also approaches zero. There are two ways to reduce the ambiguity of safety incidents for IDSM: (1) suppressing signal noise and (2) strengthening authoritative safety information.

The signal noise amount is defined to measure the effects of signal noise. The amount of signal noise received by node *i* is represented as *R_i_* (*i* may be 2, 3, …, *n*); rumor coefficient *l_i_* is defined to describe the transmission efficiency of safety information distortion between the senders and the receivers. Furthermore, similar to the safety information amount, the signal noise amount is the amount of signal noise received by the receivers; similar to the propagation coefficient, the rumor coefficient is the transmission rate of signal noise amount between the upper and lower nodes. The rumor coefficient reflects the influence degree of signal noise. Generally, the propagation coefficient of safety information between nodes is less than or equal to 1, regardless of the creativity and emergence of safety information. However, rumor coefficient *l_i_* may be much greater than 1 because safety information distortion may exhibit explosive spreading.

Similar to the safety information amount [4], the relationship between signal noise amount and rumor coefficient is shown in Equation (2). In this study, the ambiguity of safety incidents at node *i* is represented by the ratio of the signal noise amount to the safety information amount, as seen in Equation (3).

*R_i_*_+1_ = *l_i_*_+1_ × *R_i_* (2)

*A_i_* = *R_i_*/*I_i_* (3)

The meanings of the parameters in the formula are as follows: *R*_*i*+1_ is the signal noise amount of node *i* + 1; *l*_*i*+1_ is the rumor coefficient of node *i*+1; *R_i_* is the signal noise amount of node *i*; *I_i_* is the safety information amount of node *i*; *A_i_* is the safety incident ambiguity of node *i*.

## 3. Delayering Management Mode

Delayering management mode is a method of enterprise organization management, which compresses the organizational structure into a “delayering” structure by reducing the number of levels along with increasing the width [5,6]. The most important features of a delayering structure are as follows: (1) the connections of the intermediate level are increased; (2) the management span and structure density are increased; (3) and the chains and levels of organization management are shortened. In other words, this structure is characterized by “being widened and shortened”.

Delayering management mode can remedy some defects of hierarchical management mode, such as low information efficiency and long transmission time [5,6]. It has the advantage of high information efficiency and can improve the speed and accuracy of information communication. The information cluster structure can reduce the loss of information, the obstacles to information transmission, and the error and delivery time of intermediate links. It emphasizes the systematic nature and the impact of management in order to make the management structure more compact. It also forms the shortest management chain by reducing the command links.

## 4. Proposal of IDSM

The delayering mode has a high level of information efficiency, which is crucial for managing safety information. A higher information efficiency can improve the symmetry of safety information and reduce the ambiguity of safety incidents. Safety information distortion is mainly caused by the low efficiency of safety information transmission, and the delayering management mode can solve this problem. Therefore, the IDSM model is proposed to control the information distortion by taking advantage of the delayering management mode. IDSM can improve the efficiency of safety information, thereby reducing safety information distortion.

The safety information flow network resembles the organizational structure of enterprise management. The proposed safety information flow network begins with safety incidents, based on the network of interpersonal relationships. The transmission processes of safety information distortion are exhibited in a multi-level safety information flow network. Both the established safety information flow network and organizational structure have a specific starting point. Based on the above two points, IDSM is indeed a feasible method.

Certain methods of safety information flow network adjustment can be adopted to execute the delayering management mode based on graph theory [4,38]. By combining delayering management mode and graph theory, it can be seen that the network structure of IDSM has the following three characteristics: (1) Delayering mode requires fewer hierarchies in the transmission structure. In other words, it is necessary to shorten the safety information distortion chains. The nodes in the minimum control set are first adjusted due to their decisive effect on the entire network. The algorithm of graph theory is used to obtain a minimum control set; nodes in this set are upgraded to shorten their shortest paths, and they therefore have a higher rank and more accurate safety information, enabling the other nodes to receive accurate safety information. This approach is equivalent to bringing people closer to the truth of safety incidents, such as confirming the safety incidents on the scene [4]. (2) The delayering mode requires the intermediate layers of the transmission structure to have a large radiation range. On the one hand, the connectivity of the safety information chain is improved by increasing the safety information connections, which can expand the influence range of the intermediate layer. This approach is equivalent to increasing the safety information connection between people. On the other hand, the connectivity of the safety information distortion chain is weakened by removing its connections. This means that the impact scope of safety information distortion is also weakened because safety information distortion cannot be passed to other places besides the connection. This approach is equivalent to reducing the connection of safety information distortion between people. (3) The delayering mode requires a tight transmission system. In other words, the structural holes are constructed to increase the tightness of the safety information chain and the two-way communication aids in forming strong connectivity. This approach is roughly equivalent to increasing the communication of the safety incident truth between people.

In a word, IDSM is an approach to managing safety information flow for reducing safety information distortion based on the delayering mode and graph theory. It aims to accelerate the flow of safety information and curb the development of signal noise. In addition, the information superiority of delayering mode is used to manage safety information distortion in this method. IDSM is generally applicable to managing the safety information distortion of safety information flow before, during, and after the occurrence of safety incidents. The networks based on interpersonal relationships are adjusted before the spread of safety incidents, which can reduce the generation of safety information distortion to achieve pre-action management; the spreading of safety information distortion is controlled to reduce its scope and impact, which can achieve the emergency management of safety information distortion; and safety information distortion is investigated from the perspective of the causes and conditions, which can achieve after-action IDSM. The main roles of IDSM are as follows:

(1) IDSM can help to identify high-risk nodes where rumors occur, reduce safety decision-making errors caused by information distortion, and achieve the aim of incident prevention. By optimizing the safety information flow structure, IDSM can provide solid evidence for evidence-based safety management.
(2) In case of an accident, IDSM helps to quickly contain the spread of distorted information, manage the consequences of the accident, and increase the chances of making effective safety decisions.
(3) Accident investigation can be aided by examining the safety information structure, and IDSM offers a strategy for optimizing the information flow structure for crisis learning.

## 5. Implementation of IDSM

IDSM is developed by combining delayering mode and graph theory. The strategies of IDSM are divided into two aspects, based on the relationship between signal noise and safety information.

### 5.1. Methods for Signal Noise Suppression

It is necessary to limit the spread of signal noise due to its destructive nature. Methods of signal noise suppression include reducing the sources of safety information distortion, narrowing or blocking transmission channels, reducing transmission power, removing rumor nodes, removing rumor connections, splitting structural holes, removing two-way communication, and reducing the capacity of the connections.

**Figure 3:**
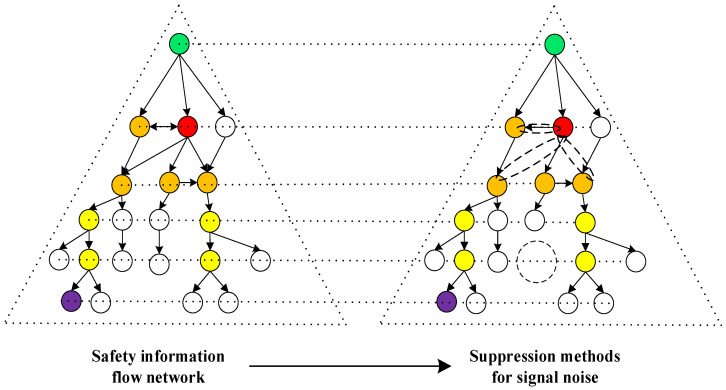
An example of a safety information flow network and its signal noise suppression methods.

To understand the signal noise suppression strategy more intuitively, an example is shown in Figure 3. In Figure 3, each individual circle represents a node of the signal noise transmission, which serves as both a receiver and sender of safety information distorted chains. A solid line connecting nodes shows the flow of safety information and signal noise, while arrows indicate the direction of transmission. A one-way arrows represent the one-way spread of rumors between nodes, while a two-way arrows represent the two-way spread of rumors between nodes. The dotted circle indicates that the content has been changed. Furthermore, the red node is the rumor node, which means the starting point of the rumor, and the rumor node is usually located through accident investigation or noise monitoring. The light green node is the central node, the purple node is the target node, the orange nodes are the suspicion nodes, and the yellow nodes are the nodes with very high importance.

The left side of Figure 3 is the safety information flow network, and the right side is the safety information flow network after executing management activities. Their main differences manifest in three aspects: (1) a rumor node is removed; (2) the transmission paths of certain rumor nodes are disconnected; and (3) the two-way communication is changed to a one-way connection.

### 5.2. Methods for Safety Information Enhancement

Safety information is the opposite of signal noise. Strengthening safety information is also a powerful way of managing safety information distortion, such as narrowing the transmission channels and reducing transmission power. Authoritative safety information, which is the truth of safety incidents, can adequately suppress false safety information distortions. Moreover, refuting nodes are set to reduce the impact and spread the scope of safety information distortion in the network. As a purifying agent, safety information can eliminate the impact and interference of signal noise; it can purify the process of safety incident transmission and create a safe environment for safety information flow. The methods of enhancing safety information include increasing refuting nodes, upgrading nodes, increasing connection (such as refuting nodes, edge nodes, and target nodes), enhancing two-way communication, establishing structure holes, and increasing the capacity of the connection.

**Figure 4:**
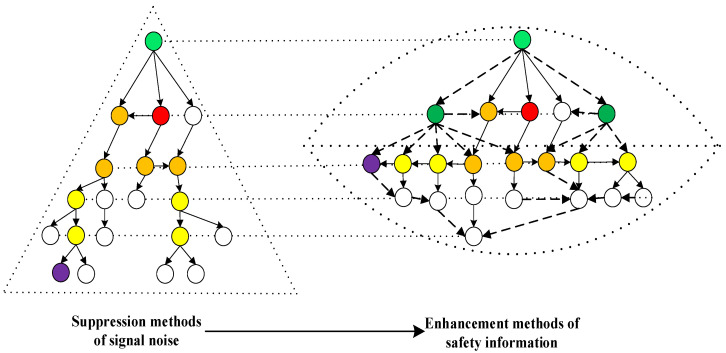
An example of a safety information flow network and its safety information enhancement methods.

In Figure 4, a dotted line indicates a newly increased connection, according to the strategy of safety information enhancement, and a green node is a refuting node. The colors of the other nodes in Figure 4 represent the same meanings as in Figure 3. The main feature of Figure 4 is the increase in the number of refuting nodes and their connections. The location of the first refuting node is connected to the suspicion node, which can narrow the scope of signal noise and prevent the spread of safety information distortion; the location of the second refuting node is connected to the important nodes in order to purify important nodes and their lower nodes, which can reduce the impact of signal noise.

The research results are as follows: (1) The initial safety information distortion is transmitted in a pyramid-shaped hierarchical structure. (2) The ideal safety information flow network is a delayering-shaped structure, which is a delayering management mode. (3) Therefore, managing safety information distortion can be realized through the ideal delayering mode of signal noise. (4) IDSM makes safety information transmission more efficient; thus, the impact of signal noise is reduced to a certain degree.

### 5.3. General Procedures for IDSM

To implement IDSM, it is necessary to calculate the amount of safety information and signal noise. Additionally, in order to control the entire information network, finding the minimum control set is necessary. IDSM can be carried out by using a directed graph algorithm and controlling the amount of safety information and signal noise at specific nodes by adjusting their connectivity and the structural hole and flow direction.

**Figure 5:**
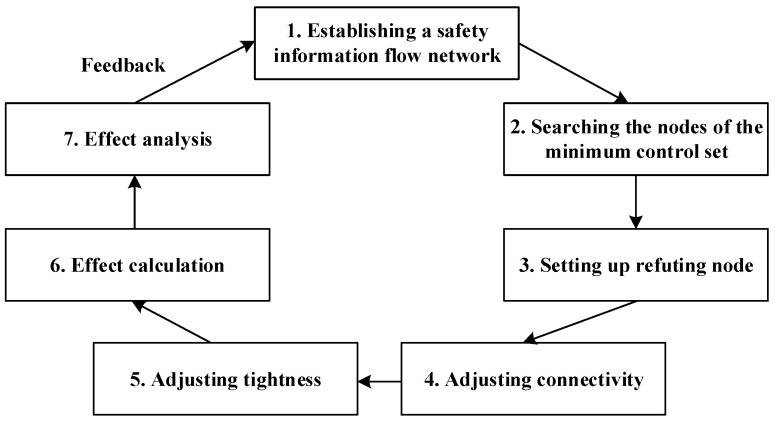
General procedures for IDSM.

A workflow diagram of the IDSM method is presented in order to clarify the general procedures for IDSM (see Figure 5) [4]:

(1) Establishing a safety information flow network: this is established based on the specific process of safety information distortion transmission.
(2) Searching the nodes of the minimum control set: the minimum control set of the safety information flow network is calculated by the algorithm of graph theory. Since rumor nodes may generate more powerful rumors based on the content of signal noise refuting, they should not be selected for the minimum control set.
(3) Setting up a refuting node: the refuting node is set as the primary node and it connects with the nodes of the minimum control set. The primary node should connect with refuting nodes because of the irreplaceability of the central node. The number of refuting nodes must be determined according to the specific radiation range of the refuting node, and the requirement is that all nodes of the minimum control set are within the reach of the radiation to ensure the effect of the refuting node.
(4) Adjusting connectivity: the safety information flow network is managed in order to reduce the connectivity based on the suppression strategies of safety information distortion. The safety information chain is managed in order to enhance connectivity based on the enhancement strategies of safety information.
(5) Adjusting tightness: the tightness of the safety information chain is strengthened based on the enhancement strategies of safety information and weakened based on the suppression strategies of safety information distortion.
(6) Effect calculation: some parameters related to safety information distortion are calculated, such as safety information amount, signal noise amount, and transmission time. The indicators of IDSM performance that can be measured are the amounts of safety information and signal noise. The optimization effect can be calculated by quantitatively comparing the amounts of safety information and signal noise before and after optimization. If safety information after optimization has a higher value than before optimization and signal noise after optimization has a lower value than before the optimization, the optimization model is considered valid. The optimization effect can also be analyzed and compared before and after management by looking at four dimensions: time, content, scope, and structure. These dimensions provide a comprehensive view of the optimization results.
(7) Effect analysis: the five dimensions are analyzed and compared in order to accurately obtain the effect of IDSM, including time, content, quantity, scope, and structure, before and after management. First, time management: if the transmission time of safety information distortion is reduced, the possibility of safety information distortion transmission can also be reduced. The survival time of safety information distortion can be compressed by accelerating the publication of safety truth. Second, content management: it is necessary to improve the ability of the public to distinguish safety information distortion and to manage safety information distortion from a content perspective. Third, quantity management: signal noise amount is controlled in order to reduce the incendiary and destructive forces of safety information distortion. Fourth, scope management: the scope of the spread of safety information distortion is controlled in order to reduce the affected population of safety information distortion. Fifth, structure management: the structure of safety information distortion is managed in order to increase the connectivity of the safety information chains and to weaken the connectivity of the safety information distortion chains.

## 6. Method Application

### 6.1. Case of Radiation Rumors in Qixian County, China

The radiation rumors in Qixian County, China [44], were selected as a specific case study of distorted safety information flow to gain a deeper understanding of the IDSM. The development process of the safety information flow is shown in Table 2.

### 6.2. Case Study Procedures

**Figure 6:**
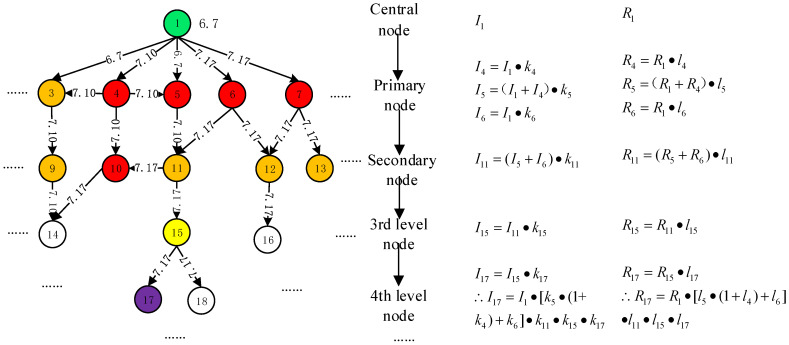
Safety information flow network of radiation rumors in Qixian County.

(1) Establishing a safety information flow network: certain research nodes are selected to construct a safety information distortion chain, and a safety information flow network is created based on the case study, as shown in Figure 6. The numbers on the connection line indicate the latest transmission time of signal noise between the two nodes because the latest signal noise can be updated and can cover previous safety information; the light green node is the central node that indicates the safety status of an irradiation plant; the green node is a refuting node, the red node is a rumor node, the orange nodes are suspicion nodes, the yellow nodes are key nodes or important nodes, the purple node is the target node; and the number *i* in the circle represents the name of the node.

(2) Searching nodes of the minimum control set: nodes of the minimum control set are obtained according to the algorithm of the directed graph [45]. First, the control matrix *X* is created and two nodes with connections are marked as 1. Second, the maximum element sum in all rows is considered to be *D*, so *D* = {*v*_1_}; then, both row *D* and the column with the element *D* are deleted, which means the 1st row and the 1st to 6th columns are removed, so *D* = {*v*_15_}; the 13th row and the columns numbered 13, 15, and 16 are removed; and the minimum control set is obtained until all the columns are removed, so *D* = {*v*_1_, *v*_9_, *v*_11_, *v*_12_, *v*_13_, *v*_15_}. Thus, nodes of the minimum control set include nodes 1, 9, 11, 12, 13, and 15.

$$\begin{array}{l} \;1\;3\;4\;5\;6\;7\;9\;10\;11\;12\;13\;14\;15\;16\;17\;18\\ X=\begin{array}{c} 1\\ 3\\ 4\\ 5\\ 6\\ 7\\ 9\\ 10\\ 11\\ 12\\ 13\\ 14\\ 15\\ 16\\ 17\\ 18 \end{array}\left[\begin{array}{cccccccccccccccc} 1 & 1 & 1 & 1 & 1 & 1 & 0 & 0 & 0 & 0 & 0 & 0 & 0 & 0 & 0 & 0\\ 0 & 1 & 0 & 0 & 0 & 0 & 1 & 0 & 0 & 0 & 0 & 0 & 0 & 0 & 0 & 0\\ 0 & 1 & 1 & 1 & 0 & 0 & 0 & 1 & 0 & 0 & 0 & 0 & 0 & 0 & 0 & 0\\ 0 & 0 & 0 & 1 & 0 & 0 & 0 & 0 & 1 & 0 & 0 & 0 & 0 & 0 & 0 & 0\\ 0 & 0 & 0 & 0 & 1 & 0 & 0 & 0 & 1 & 1 & 0 & 0 & 0 & 0 & 0 & 0\\ 0 & 0 & 0 & 0 & 0 & 1 & 0 & 0 & 0 & 1 & 1 & 0 & 0 & 0 & 0 & 0\\ 0 & 0 & 0 & 0 & 0 & 0 & 1 & 0 & 0 & 0 & 0 & 1 & 0 & 0 & 0 & 0\\ 0 & 0 & 0 & 0 & 0 & 0 & 0 & 1 & 0 & 0 & 0 & 1 & 0 & 0 & 0 & 0\\ 0 & 0 & 0 & 0 & 0 & 0 & 0 & 0 & 1 & 0 & 0 & 0 & 1 & 0 & 0 & 0\\ 0 & 0 & 0 & 0 & 0 & 0 & 0 & 0 & 0 & 1 & 0 & 0 & 0 & 1 & 0 & 0\\ 0 & 0 & 0 & 0 & 0 & 0 & 0 & 0 & 0 & 0 & 1 & 0 & 0 & 0 & 0 & 0\\ 0 & 0 & 0 & 0 & 0 & 0 & 0 & 0 & 0 & 0 & 0 & 1 & 0 & 0 & 0 & 0\\ 0 & 0 & 0 & 0 & 0 & 0 & 0 & 0 & 0 & 0 & 0 & 0 & 1 & 0 & 1 & 1\\ 0 & 0 & 0 & 0 & 0 & 0 & 0 & 0 & 0 & 0 & 0 & 0 & 0 & 1 & 0 & 0\\ 0 & 0 & 0 & 0 & 0 & 0 & 0 & 0 & 0 & 0 & 0 & 0 & 0 & 0 & 1 & 0\\ 0 & 0 & 0 & 0 & 0 & 0 & 0 & 0 & 0 & 0 & 0 & 0 & 0 & 0 & 0 & 1 \end{array}\right] \end{array}\begin{array}{l} \;9\;10\;11\;12\;13\;14\;15\;16\;17\;18\\ X^{\prime }=\begin{array}{c} 3\\ 4\\ 5\\ 6\\ 7\\ 9\\ 10\\ 11\\ 12\\ 13\\ 14\\ 15\\ 16\\ 17\\ 18 \end{array}\left[\begin{array}{cccccccccc} 1 & 0 & 0 & 0 & 0 & 0 & 0 & 0 & 0 & 0\\ 0 & 1 & 0 & 0 & 0 & 0 & 0 & 0 & 0 & 0\\ 0 & 0 & 1 & 0 & 0 & 0 & 0 & 0 & 0 & 0\\ 0 & 0 & 1 & 1 & 0 & 0 & 0 & 0 & 0 & 0\\ 0 & 0 & 0 & 1 & 1 & 0 & 0 & 0 & 0 & 0\\ 1 & 0 & 0 & 0 & 0 & 1 & 0 & 0 & 0 & 0\\ 0 & 1 & 0 & 0 & 0 & 1 & 0 & 0 & 0 & 0\\ 0 & 0 & 1 & 0 & 0 & 0 & 1 & 0 & 0 & 0\\ 0 & 0 & 0 & 1 & 0 & 0 & 0 & 1 & 0 & 0\\ 0 & 0 & 0 & 0 & 1 & 0 & 0 & 0 & 0 & 0\\ 0 & 0 & 0 & 0 & 0 & 1 & 0 & 0 & 0 & 0\\ 0 & 0 & 0 & 0 & 0 & 0 & 1 & 0 & 1 & 1\\ 0 & 0 & 0 & 0 & 0 & 0 & 0 & 1 & 0 & 0\\ 0 & 0 & 0 & 0 & 0 & 0 & 0 & 0 & 1 & 0\\ 0 & 0 & 0 & 0 & 0 & 0 & 0 & 0 & 0 & 1 \end{array}\right]\ldots \ldots \end{array}$$

**Figure 7:**
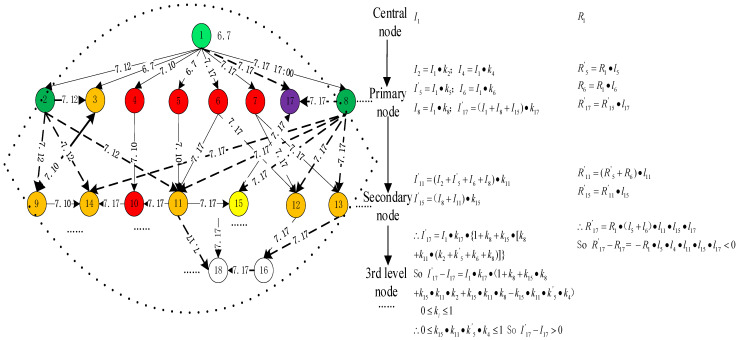
IDSM of radiation rumors in Qixian County, China.

(3) Setting up refuting nodes: some refuting nodes have been added, such as node 2 on 12 July and node 8 at 5:00 pm on 17 July. Nodes of the minimum control set should connect to refuting nodes. Therefore, nodes 2 and 8 are connected to nodes 1, 9, 11, 12, 13, and 15, which is equivalent to upgrading node 15.

(4) Adjusting connectivity: some safety information distortion chains are removed, such as the connection between nodes 3 and 4 as well as 4 and 5. Some information chains are increased, such as the connection between nodes 2 and 8 as well as 3 and 14. Increasing the dissemination of safety information can expand the scope of safety information flow and is beneficial to the management of safety information distortion. The edge nodes are connected. The target node is upgraded by connecting to the central node and the refuting node.

(5) Adjusting tightness: the edge nodes are constructed as structural holes, such as nodes 12, 13, and 16. A two-way communication of safety information is established between nodes, such as nodes 3 and 9. Safety information distortion structure holes where connections 3–4 and connections 4–5 are located are dismantled.

(6) Effect calculation: according to Figure 6, the adjusted network structure is obtained according to the management strategies of safety information distortions, as shown in Figure 7. The colors of the other nodes in Figure 7 represent the same meanings as in Figure 6. It is assumed that the propagation coefficient and rumor coefficient of nodes are unchanged before and after adjustment. Moreover, it is assumed that the time of receiving safety information distortions for each node is unchanged before and after adjustment and signal noise and safety information can be transmitted based on interpersonal relationships. The changes in the safety information amount and signal noise amount are calculated based on Formula (2), as shown in Figure 7. The difference between signal noise amount and safety information amount is that the rumor node has safety information amount, while the refuting node has no signal noise amount.

(7) Effect analysis: the management effect of safety information distortions can be seen according to the comparison between Figure 6 and Figure 7:

- Time aspect: target node 17 is connected to the central node. The real information transmission time of the target node is advanced from the 17 July to the 12 July, which reduces the impact of safety information distortions on the target node. Similarly, the time when some nodes receive real safety information is advanced to 12 July, such as nodes 9, 14, 11, 15, 18, and 16; their safety information reception time is the same as refuting node 2. Therefore, the time of safety information reception is advanced in the transmission process of safety information distortions.
- Content aspect: after setting up the refuting node, the propagation coefficient of safety information increases because of the high credibility and authority of the refuting node. In addition, the public’s belief in signal noise decreases due to the predation effect of safety information on signal noise. Therefore, the rumor coefficient decreases. Safety information concerning safety incidents is generally recognized and accepted based on the calculation result of Figure 7.
- Quantity aspect: as shown in Figure 7, the safety information amount increases while the signal noise amount decreases after IDSM. Therefore, the ambiguity of safety incidents and the risk of safety information distortions also decrease.
- Scope aspect: the number of nodes in all safety information distortion chains has been reduced from 16 to 5, which means that the scope of safety information distortions is narrowed.
- Structure aspect: the network before adjustment is a tree-like hierarchical structure. The adjusted network is a delayering structure. The safety information flow network has been cut from 5 layers to 4 layers. The connectivity of the safety information chains increases and that of safety information distortion chains is weakened.

The following conclusions can be drawn from the method’s application: firstly, DISM can help determine the scheme to optimize the information flow structure and reduce information distortion. Secondly, the effectiveness of the DISM optimization scheme can be clarified as the scheme improves in five aspects, including time, content, quantity, scope, and structure. IDSM can realize the control of safe information flow and can reduce the adverse consequences caused by signal noise. Finally, the adjustment of the safety information flow structure has been shown to be beneficial in enhancing system safety. In the era of big data, information distortion management can be realized through the delayering management of the safety information flow based on graph theory. It can effectively reduce information distortion from safety information and signal noise. In conclusion, IDSM is a feasible approach to achieving effective safety management by the management of information distortion, which controls the flow of both safety information and signal noise.

## 7. Discussion and Conclusions

### 7.1. Discussions

This study proposes a new method of IDSM based on the delayering mode and realizes IDSM through graph theory. It leverages the information superiority of the delayering structure to limit the growth of safety information distortion. IDSM can effectively improve the accuracy of safety information flow and reduce the distortion of safety information flow. While IDSM has high flow rates, interaction rates, contact rates, and transmission rates regarding safety information, it has the low flow rates, interaction rates, contact rates, and transmission rates in terms of signal noise. This study reinforces the notion that safety information plays a crucial role in maintaining system safety and presents a novel approach to guarantee the reliability of information for accident control. The method of safety information and organization management is applied to the field of IDSM, which can reduce the threat of safety information distortion to system safety and promote effective emergency response.

Firstly, based on Lei’s focus on the improvement of safety information efficiency [4] and Chen’s emphasis on negative signal noise [3], this approach considers two paths of controlling information distortion, including safety information and signal noise simultaneously, which can improve management outcomes and control accidents caused by information distortion more effectively.

Secondly, this paper focuses on the impact of information structure on the amount of safety information, rather than just the means and ability to search for information in evidence-based safety management [32]. Instead of only controlling information distortion from the source, receiver, and channel in risk information communication, the paper proposes the IDSM method, which takes into account the coupling relationship between these three factors and optimizes the information flow process through a delayering management model. The paper suggests that the distribution of safety information can affect the distribution of risks and that the IDSM method can provide the most reasonable and effective safety information distribution under limited conditions.

Finally, this paper not only proposes the IDSM method but also provides an implementation strategy based on graph theory, making it more practical than evidence-based safety management [32] and precise safety management [36], which remain at the theoretical level. By bridging the gap between theory and practice, the IDSM implementation strategy is a useful guide for reducing information distortion. Its high feasibility and obvious effectiveness make it a practical solution for improving safety management.

However, there are still some limitations: the IDSM method demands safety incidents to have clear rumor nodes and transmission times, which necessitates a significant requirement for information collection regarding the development of safety information distortion. If safety information distortion occurs in a dynamic network with a large number of nodes, the calculation process of dynamic safety information amount and signal noise amount is cumbersome. Computer programs can be developed to automate the analysis and adjustment of safety information distortion chains, making IDSM more efficient and dynamic. In addition, this study simplifies certain real situations, and future research can further solve such problems. For example, this study excludes the self-reproduction of safety information distortion and the positive emergence of safety information. Although the safety information flow network is based on the connectivity of graph theory, it is not comprehensive enough to take full control of safety information distortion. Future research can continue to use graph theory for more a scientific IDSM.

### 7.2. Conclusions

This study proposes a set of new concepts on safety information distortion. The predator relationship between safety information and signal noise is clarified. Signal noise suppression and safety information enhancement are two implementation paths of IDSM.

This study provides a new methodology for improving IDSM and ensuring system safety; IDSM is proposed and implemented based on graph theory. IDSM is suitable for the safety management process to solve information distortion. Adjusting the safety information dissemination structure to achieve a delayering model can effectively improve system safety. The ideal transmission structure of safety information distortion is a delayering one. The minimum control set of the directed graph algorithm is used to realize the whole network management of safety information distortion; the amount of safety information and signal noise for specific nodes are controlled by adjusting the connectivity and safety information distortion control is achieved by modifying the structural hole and communication direction. Furthermore, the effect of IDSM can be measured in terms of time, content, quantity, scope, and structure. Evidence from a case study showed that the flow of signal noise is reduced based on a case study, while the flow of safety information is increased.

## Figures and Tables

**Table 1:** Related concepts applied for safety information distortion study.

No.	Concept	Definition
1	Rumor node	The rumor node refers to the starting node generated by safety information distortion.
2	Refuting node	The refuting node is the opposite of the rumor node; its information is safety information, which reflects real safety incidents. The setting of refuting node has certain requirements for its form, such as official media, authoritative institutions, government speech, and expert analysis. Consequently, refuting node information has high credibility and authority, enabling the public to distinguish safety truth from safety information distortion.
3	Rumor chain	A rumor chain refers to the chain where the rumor node is located; nodes in a rumor chain are affected by safety information distortion. Therefore, it is necessary to purify the rumor chain to reduce the scope of safety information distortion.
4	Suspicion node	The suspicion node follows the rumor node in a rumor chain. It is most susceptible to rumor nodes. Therefore, the suspicion node is the focus of safety information management to reduce the reach of safety information distortion.
5	Structural hole	A structural hole [41] refers to the nodes that form a closed loop, forming a relatively compact structure. The nodes in the structural hole have the advantage of information resources, they communicate closely, and their information transmission speed is relatively fast.
6	Edge node	The edge node is at the end of a safety information distortion chain; it has few sources of safety information and is therefore susceptible to safety information distortion.
7	Connectivity	Connectivity [38] reflects the tightness of nodes; it is an important parameter of network complexity and IDSM. Connectivity analysis mainly discusses whether multiple points specified are connected. This study adjusts the connectivity to achieve IDSM. The aim is to create a connected graph of the safety information chain that allows safety information to spread throughout the entire network. If the safety information distortion chain becomes a separate graph, safety information distortion is trapped in a small area of the entire network.
8	Control set	This selected vertex set in a connected graph can control all other vertexes.
9	Minimum control set	A minimum control set [38] refers to a control set with the smallest number of vertexes.

**Table 2:** Case analysis of the development process of safety information distortion in Qixian County, China.

No.	Time	Event	Detail
1	7 June 2009	A safety incident occurred	While an irradiation plant in Qixian County, Henan Province, China, was irradiating pepper, the shelf of the radioactive source tilted due to the collapse of the cargo, and the radioactive source could not be properly lowered into the source-storing well. The pepper spontaneously ignited because of the prolonged exposure to radiation heat.
2	10 July	Safety information distortion began	A post titled “Cobalt-60 Leakage in Qixian County” was widely circulated, saying that “the radioactive source of a local irradiation plant cannot be placed into deep underground cooling water, causing the cobalt-60 to leak”. The post caused panic among the masses.
3	12 July	The government refuted the rumor	The local government held a press conference to explain the situation, announcing that there was no such pollution problem caused by radiation source leakage.
4	11:00 a.m. on 17 July	Safety information distortion intensified while the safety incident was being handled	The officials of the environmental protection department and experts took action to remove the radioactive sources using robots. The robots were damaged by accident. However, safety information distortion emerged, claiming that “the irradiation plant was going to explode and the robot had been burned by radiation heat”. At noon, many residents left the county to seek “refuge” because of the explosion rumors.
5	5:00 p.m. on 17 July	The government refuted the rumors from multiple aspects	The local government persuaded the local people to return home by releasing the facts to local people through newspapers, radio stations, TV stations, mobile phones, etc.
6	19 July	Safety information distortions calmed down	The police announced a successful arrest of five rumormongers of the Cobalt-60 incident in Qixian County.

## Data Availability

Restrictions apply to the availability of these data. The case in this paper was obtained from literature [44]. This paper primarily examines the development process of the case through an analysis of its information structure.
